# Determinants of unmet need for family planning among currently married reproductive age women at Dewa Chefa District of Oromia special zone, Amhara region, Ethiopia, 2021; a case-control study

**DOI:** 10.1186/s12905-024-02939-x

**Published:** 2024-02-08

**Authors:** Mohammed Ammie, Mastewal Arefaynie, Bezawit Adane, Kedir Hussein, Mohammedsani Hassan

**Affiliations:** 1 The Carter Center Ethiopia, Oromia Special Zone of Amhara Region, Kemissie, Ethiopia; 2 School of Public Health, College of Medicine and Health Sciences, Wollo University, Dessie, Ethiopia; 3 Department of Epidemiology and Biostatistics, College of Medicine and Health Sciences, Injibara University, Injibara, Ethiopia; 4 Dubti Hospital of Afar Regional State, Dubti, Ethiopia; 5 The Carter Center Ethiopia, North Shoa Zone of Amhara Region, Debre Birhan, Ethiopia

**Keywords:** Unmet need, Family planning, Fecund, Reproductive age woman, Kemissie

## Abstract

**Background:**

Unmet need for family planning is a proportion of women among reproductive age group who want to stop or delay childbearing but are not using any method of contraception. One in ten married women face unmet need for family planning world-wide whereas, one in five women in Africa. Thus, by understanding factors associated with unmet need specific to the study area; the study contributes to planning and intervention of programs, gives additional finding for controversies in earlier studies, and also helps as a baseline for other researchers conducting studies on similar topics.

**Methods:**

A community-based unmatched case-control study was conducted from March 29-April 25, 2021 G.C on 462 currently married reproductive age women (154 cases and 308 controls) in Dewa Chefa District. Currently married reproductive-age women who were fecund, and wanted to limit or delay childbearing but were not using any contraceptive methods were taken as cases and currently married reproductive-age women who were using family planning or did not want to use were taken as controls. A structured and pre-tested questionnaire was used to collect data. Collected data were entered into Epi-data 3.1 and exported to SPSS 23 for analysis. Binary Logistic regression was conducted and variables with p-value < 0.05 were taken as statistically significant.

**Results:**

A total of 462 women participated in this study, with 100% response rate. The mean age of the respondents was 27.92 years (with SD of ± 6.3) Age of woman 35–49 [AOR = 6.6 (1.1–39)], having poor knowledge on family planning [AOR = 1.9 (1.1–3.1)], using family planning decided by husband [AOR = 3.8 (2.1–6.9)], using family planning decided together [AOR = 2.3 (1.07–5.1)] and have no support and disapproval of husband for family planning use [AOR = 2.1 (1.08-4)] were factors significantly associated with unmet need.

**Conclusion and recommendations:**

Age of the woman, main decider of family planning use, knowledge about family planning and support and approval of spouse for family planning use were found to have significant association with unmet need for family planning. Thus, family planning providers, District health office, and other concerned bodies should strengthen female empowerment and male involvement in the program with strong couple counseling to reduce unmet need.

## Introduction

Unmet need for family planning is a proportion of women who want to stop or delay childbearing but are not using any method of contraception, reported as a percentage with reference to all women of reproductive age (15–49 years) [1]. Married women of reproductive age are said to have unmet need if they are fecund, do not want a child in the next 2 years or at all, and are not using any method of contraception, either modern or traditional [2]. Pregnant or amenorrhea women are also considered to have unmet need if their pregnancy was mistimed or wanted no more children [3].

Ensuring access to voluntary family planning methods has various health, economic, social, and environmental benefits. It saves the lives of women and children, improves the quality of life for all and reduces morbidity and mortality from pregnancy, reduces infant deaths by as much as a fifth, relieves pressures from rapid population growth, and reduces strain on community resources (health care, education, and agriculture), and lessens pressure on the socio-political system. Evidence suggests that FP interventions contributed to more than a 25% reduction in the maternal mortality ratio [4, 5]. Serving all women in low-income countries that currently have an unmet need for modern methods would prevent an additional 54 million unintended pregnancies, including 21 million unplanned births, 26 million abortions (of which 16 million would be unsafe), and 7 million miscarriages; this would also prevent 79,000 maternal deaths and 1.1 million infant deaths [13]. Ethiopia is currently working towards reducing unmet need for FP from 22% in 2016 to 10% by the end of 2020 year [6, 8].

One in ten married women faces unmet need for family planning worldwide whereas, one in five women in Africa [9]. In 2019, 42 countries, including 23 in sub-Saharan Africa, still had levels of demand satisfied by modern methods below 50%, including three countries of sub-Saharan Africa with levels below 25% [1]. Despite recent progress in decreasing its prevalence, it is estimated that 222 million women in low- and middle-income countries have an unmet need for modern contraception [10, 11]. In our country, despite use of modern contraceptive has increased, still 22% of currently married women have an unmet need for family planning [12].

Different literatures reported that different factors influencing unmet need of family planning were age, age of marriage, religion, wealth, occupation, level of parity, number of children, educational status of self and partner, knowledge of contraceptive methods, discussion with partner and health workers, visit to health facility, partners attitude, social pressures and contraception related factors (like; availability, accessibility, affordability, side effects) [14, 16, 18, 20, 22–30]. But there are controversies between the studies, as some variables found significant in some studies are not significant by other studies.

The review of the literature shows a high prevalence of unmet need for FP in Ethiopia and determinants vary across the regions. Even though many studies were conducted on unmet need for FP, most of them were in towns/urban settings and were cross-sectional studies [17–22], which do not show the cause-effect relationship well compared to case-control studies. On the other hand, Dewa Chefa district is a rural and populous, predominantly Muslim community (where high fertility, polygamy, and widow inheritance are common) [31]. These factors lead women to have less freedom and power to decide on utilization of FP [19, 23]. However, clear evidence of unmet need for FP is not available in Dewa Chefa district.

Hence no study has been conducted on the issue in the study area, this study provides evidence on determinants of unmet need for FP from a rural setting, identifies which ones are more important to the study area, and assesses the freedom women have to decide on FP use as well. Such locally available evidence is essential for program managers and health administrators working at different levels to the formulation, planning, interventions, delivery of quality FP services, and reducing unmet need, which in return improve women’s health and reduces maternal mortality.

## Methods and materials

### Study area, period, design and populations

Community-based unmatched case control study was conducted to identify determinants of unmet need for family planning from March 29 – April 25, 2021. The study was conducted at Dewa Chefa District of Oromia special zone, Amhara region, Ethiopia. Dewa Chefa is the largest District of Oromia zone with a total population of 161,488 by 2021, of which 32,669 are reproductive age (15–49 years) women. The population is distributed into 26 kebeles. There are 52 governmental and private health facilities serving the population (7 health centers, 27 health posts and 18 private health facilities). All governmental health facilities and private clinics give family planning services [31].

**Source population**: Currently married reproductive age women in Dewa Chefa District.

**Study population**:

**Cases**: Currently married reproductive age women in selected kebeles, who were fecund, wanted to limit or delay childbearing but were not using any contraceptive methods.

**Controls**: Currently married reproductive age women in selected kebeles, who were using family planning and did not want to use.

**Inclusion criteria**:

**Cases**: Currently married reproductive age women in selected kebeles who were fecund, wanted to limit or delay childbearing but were not using any contraceptive methods; whose permanent residence were in the study area were included.

**Controls**: Currently married reproductive age women in selected kebeles who were using family planning and did not want to use; whose permanent residence were in the study area were included.

**Exclusion criteria**:

**Cases**: Currently married reproductive age women in selected kebeles who were fecund, wanted to limit or delay childbearing but were not using any contraceptive methods; who were seriously ill and unable to respond were excluded.

**Controls**: Currently married reproductive age women in selected kebeles who were using family planning and did not want to use; who were seriously ill and unable to respond were excluded.

### Sample size determination and sampling procedure

The sample size was determined by Epi info-7.2.2.6, considering predictor variables from previous studies conducted in Ethiopia [22–25]. Discussion on FP with partner was used as reference predictor for selecting the maximum sample size. With odds ratio of 1.83 and 42.5% of exposure in controls for the reference predictor and assumption of 80% power, 95% confidence interval, 10% non-response rate and a case to control ratio of 1:2, the total sample size was 462 (154 cases and 308 controls).

To get the final sample size, from the total 26 kebeles in the District, 50% of kebeles (which was 13) was chosen by lottery method from the list of all kebeles in the District. Then, with preliminary survey conducted house to house, 5154 and 10,370 women were listed as cases and controls respectively. Finally, systematic random sampling technique was employed to select the samples (currently married RAW) from the sampling frame prepared with preliminary survey at sampled 13 kebeles. The first sample was selected by lottery method in both case and control groups. Then, by determining K^th^ value for both case and control groups, cases were selected at 33th interval and controls were selected at 34th interval. The first respondent was #9 for the cases and #20 for controls, which were chosen by lottery method and were included in the study.

### Operational definition

**Currently married reproductive age group women**: Women of reproductive age group (15–49 years) who are married.

**Unmet need for FP**: a proportion of women among reproductive age group who want to stop or delay childbearing but are not using any method of contraception.

**Knowledge about FP**: Each knowledge question answered correctly was scored one point while question answered incorrectly was scored zero. The total score ranging from 0 to 16 obtained by each respondent was added up and the mean score was computed to categorize knowledge.

**Good knowledge**: when a woman correctly answered above the mean score of knowledge questions administered (i.e. greater than 7) [24].

**Poor knowledge**: when a woman correctly answered mean and below the mean score of knowledge questions administered (i.e. less than or equal to 7) [24].

### Data collection tool, procedures and quality control measures

Data was collected using interviewer administered structured questionnaire. The tool was prepared from different variables found significant from different literature and knowledge related questions were adopted from study conducted at Cameroon [32]. The variables in the questionnaire were categorized in to 3 groups (Socio demographic and socio economic related, reproductive related and knowledge and decision related questions). The questionnaire was translated from English to Amharic and Afan Oromo languages and translated back to English to check for consistency. The data was collected by 9 HW’s and 9 HEW’s who are trained on family planning and supervised by 4 officers from District health office. The data were collected house to house from the respondents as per the sampling procedure.

The questionnaire was pre-tested on 23 reproductive age women (5% of the respondents) out of study area at Kachur kebele of Kemissie town and was checked and corrected for ambiguous questions. For data collection, data collectors were trained for two days on the data collection tool and procedure and familiarized with the objective and the method of the research. The data collection process was being monitored for consistency, completeness and other issues by supervisors and principal investigator daily.

### Data analysis and processing

The collected data were coded and entered in to Epi-data 3.1 and then exported to SPSS version 23 for analysis. Descriptive statistics was done using frequency and summary measures. Binary logistic regression model was employed to identify factors associated with the dependent variable. Variables with p-value of < 0.25 were entered to multivariable logistic regression model to identify independent predictors. With all regression assumptions fulfilled [i,e: model prediction of variables improved from the beginning block (from 66.7 to 72.5%), better prediction of variables than constant only model (omnibus test of coefficients at *p* < 0.001), the dependent variable is well explained by the independent variables in the model summary and good model fitness with Hosmer and Lemeshow test (at *p* = 0.138)] and variables with p-value < 0.05 were taken as statistically significant. Multi-collinearity was checked for all variables entered to the model.

## Results

### Socio demographic and socio-economic characteristics of respondents

A total of 462 women participated in this study, with 100% response rate. The mean age of the respondents was 27.92 years (with SD of ± 6.3), with the highest proportion falling in 20–34 age category for both cases and controls. Majority of the cases (85.7%) and the controls (78.9%) lived in rural setting. More than 90% of both the cases and controls were Muslim religion followers and again 76% of both the cases and controls were ethnically Oromo. More than 119 (75%) and 196 (60%) of the study respondents in the case and control groups were didn’t attended formal education, while above 126 (80%) and nearly 216 (70%) of the partners in case and control groups respectively were didn’t attended formal education too. The mainly mentioned occupation category of the respondents were house wife (61% for cases and 49% for controls) and above half of both the cases and controls of this study had more than 5 household members. Majority of the cases 128 (83.1%) and the controls 258 (83.8%) had media source in their houses, mainly radio followed by television and mobile phone (Table 1).

### Reproductive history of respondents and unmet need for family planning

During survey period, 99 women (31.2% of cases and 16.6% of controls) were pregnant from which about half were reported as mistimed pregnancies. More than two third of respondents in both the case and control groups had previously used FP method. More than half (51.9%) of the cases and 201 (65%) of the controls had married at the age of 18 and above, from which above two third of respondents in both groups had been married only once. Above 70% respondents in both groups had less than 5 children. More than 90% of the respondents in each group intended to have more than 4 children in life (Table 2).

**Fig. 1:**
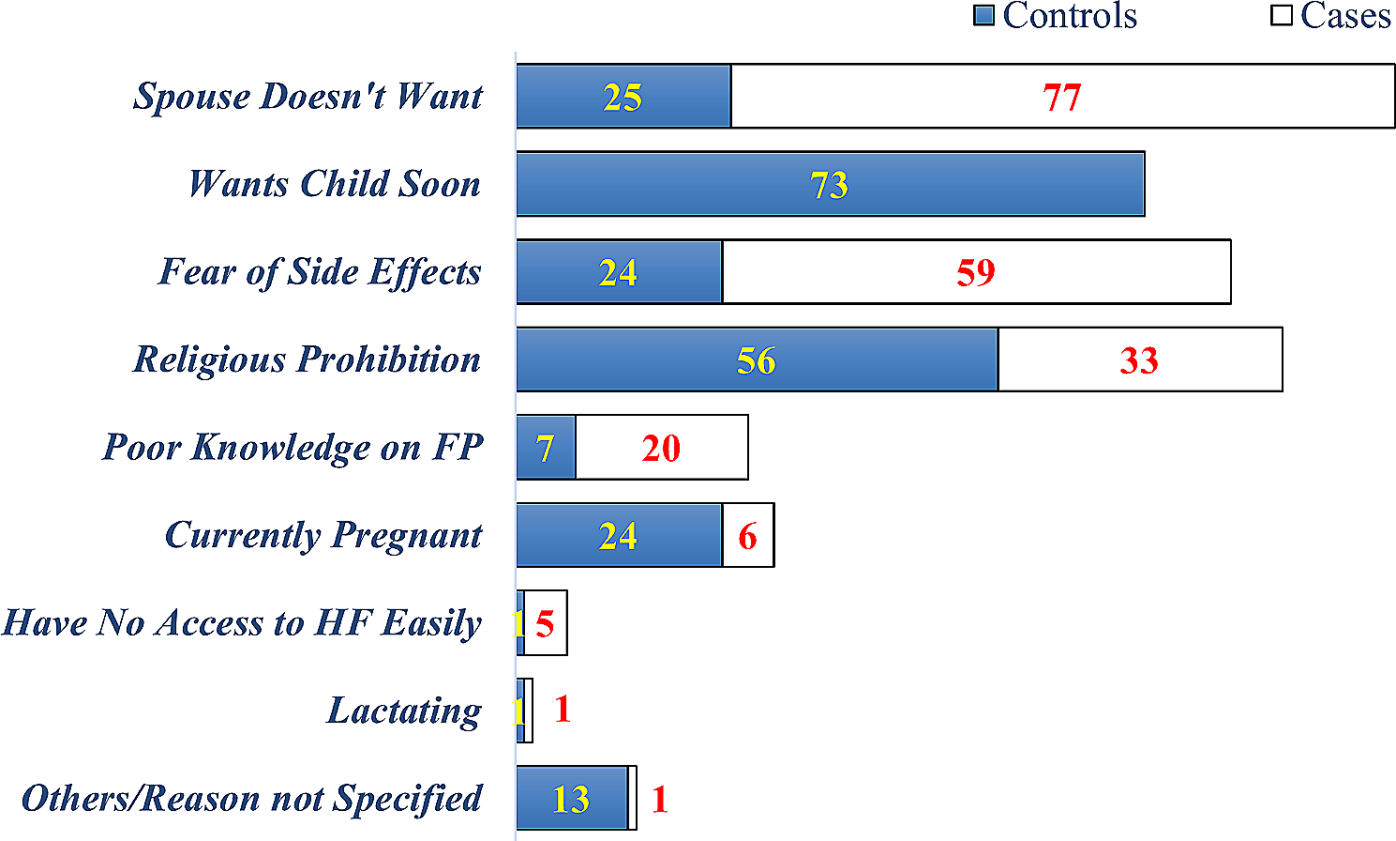
Reasons for not using FP method among reproductive age women in Dewa Chefa district, Ethiopia, March 29-April 25, 2021

Majority of the cases were not using FP method for their husband doesn’t wanted them to use, accompanied with fear of side effects and religious prohibition. While, wanting child soon (within 2 years) and religious prohibition were mainly mentioned reasons not to use FP method among the controls (Fig. 1).

**Table 1:** Socio demographic and socio-economic characteristics of reproductive age women in Dewa Chefa district, Ethiopia, March 29-April 25, 2021

Variables	Response Category	Cases n (%)	Controls n (%)
Age	< 20 years 20–34 years 35–49 years	2 (1.3%) 115 (74.7%) 37 (24%)	45 (14.6%) 208 (67.5%) 55 (17.9%)
Residence	Semi-Urban Rural	22 (14.3%) 132 (85.7%)	65 (21.1%) 243 (78.9%)
Religion	Muslim Orthodox Others	145 (94.2%) 6 (3.9%) 3 (1.9%)	287 (93.2%) 14 (4.5%) 7 (2.2%)
Ethnicity	Oromo Amhara Others	117 (76%) 33 (21.4%) 4 (2.6%)	234 (76%) 71 (23.1%) 3 (0.9%)
Respondents Educational Status	No formal education Read and write Primary Secondary College & Above	73 (47.4%) 46 (29.9%) 18 (11.7%) 6 (3.9%) 11 (7.1%)	106 (34.4%) 90 (29.2%) 43 (14%) 28 (9.1%) 41 (13.3%)
Partners Educational Status	No formal education Read and write Primary Secondary College & Above	67 (43.5%) 59 (38.3%) 12 (7.8%) 3 (1.9%) 13 (8.4%)	104 (33.8%) 112 (36.4%) 24 (7.8%) 17 (5.5%) 51 (16.6%)
Respondents Occupation	House wife Farmer Merchant Student Daily Laborer Gov’t employee Private employee	95 (61.7%) 21 (13.6%) 22 (14.3%) 2 (1.3%) 3 (1.9%) 10 (6.5%) 1 (0.6%)	152 (49.4%) 37 (12%) 33 (10.7%) 47 (15.3%) 4 (1.3%) 29 (9.4%) 6 (1.9%)
Household Members	< 5 5 and Above	58 (37.7%) 96 (62.3%)	146 (47.4%) 162 (52.6%)
Media Source Availability	Yes No	128 (83.1%) 26 (16.9%)	258 (83.8%) 50 (16.2%)
Type of Media Source Available (*n* = 386)	Television Radio Mobile Phone	36 (28.1%) 47 (36.7%) 45 (35.2%)	88 (34.1%) 102 (39.5%) 68 (26.4%)

**Table 2:** Reproductive history of reproductive age women in Dewa Chefa district, Ethiopia, March 29-April 25, 2021

Variables	Response Category	Cases n (%)	Controls n (%)
Currently Pregnant?	Yes No	48 (31.2%) 106 (68.8%)	51 (16.6%) 257 (83.4%)
Previous Use of FP method	Yes No	108 (70.1%) 46 (29.9%)	214 (69.5%) 94 (30.5%)
Age at First Marriage	< 18 years 18 and above	74 (48.1%) 80 (51.9%)	107 (34.7%) 201 (65.3%)
Repetition of Marriage	Once Twice More than Twice	111 (72.1%) 32 (20.8%) 11 (7.1%)	236 (76.6%) 64 (20.8%) 8 (2.6%)
Number of Living Children	0 1–2 3–4 5 and above	5 (3.2%) 56 (36.4%) 51 (33.1%) 42 (27.3)	56 (18.2%) 112 (36.4%) 70 (22.7%) 70 (22.7%)
Number of Intended Children in life	≤ 3 4–6 > 6 Not Decided	6 (3.9%) 64 (41.6%) 76 (49.4%) 8 (5.2%)	12 (3.9%) 132 (42.9%) 157 (51%) 7 (2.3%)

**Fig. 2:**
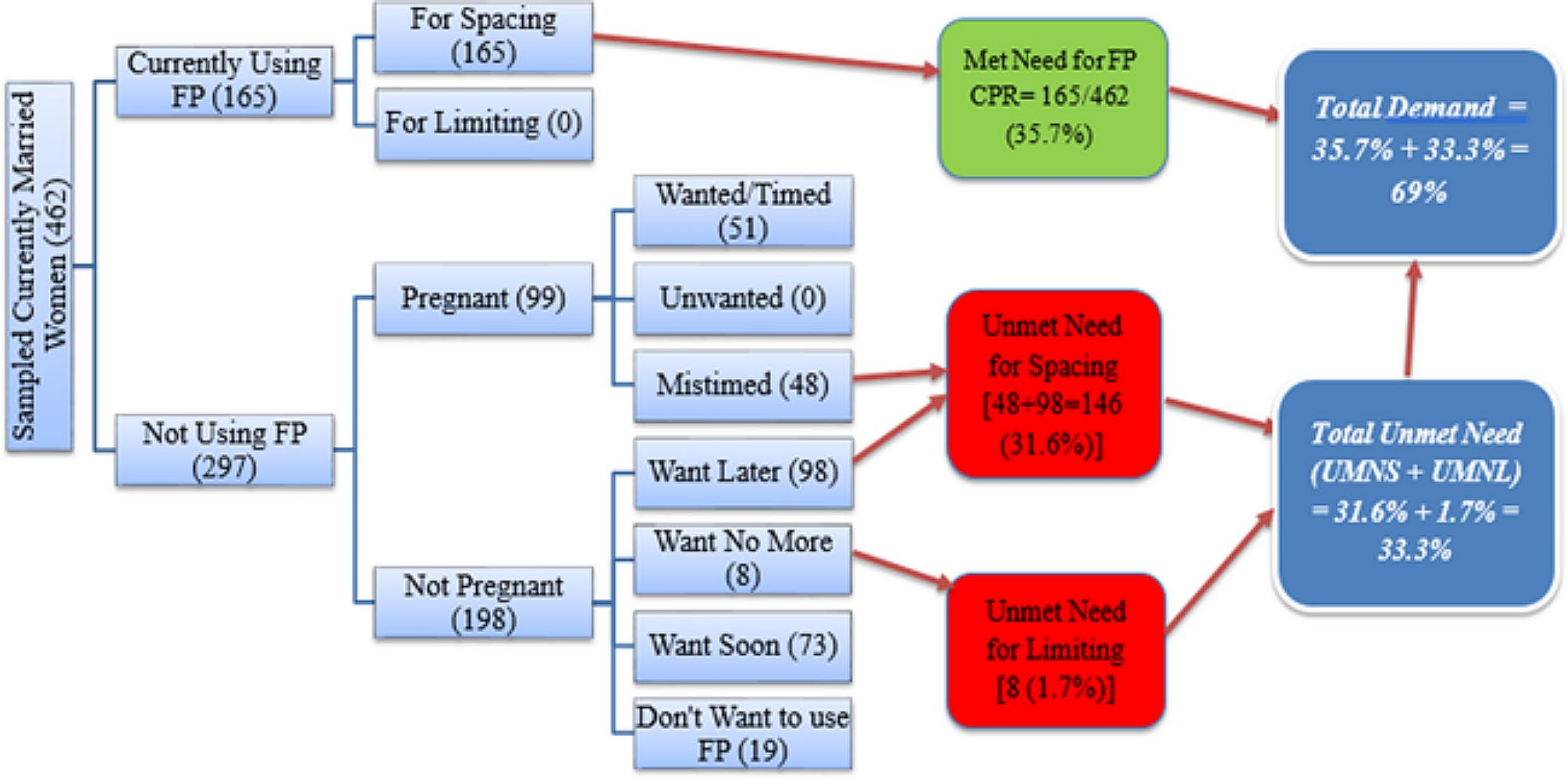
Graphical presentation of unmet need for FP in Dewa Chefa district, Ethiopia, March 29-April 25, 2021

Total demand for family planning among the respondents was found to be 69%, from which satisfied demand is only 51.7%. Most of the respondents (31.6%) had unmet need for spacing, while only 1.7% of the respondents had unmet need for limiting (Fig. 2).

### Knowledge of respondents about family planning

Almost all (99%) of the respondents in both groups had heard about family planning, from which 135 (87%) of the cases and 274 (89%) of the controls stated health professionals as major source of information for FP. Using the mean average score of respondent’s response on knowledge related questions as measurement, only 45 (29%) of the cases and 169 (55%) of the controls had good knowledge about family planning (Table 3).

**Table 3:** Knowledge of reproductive age women about FP in Dewa Chefa district, Ethiopia, March 29-April 25, 2021

Variables	Response Category	Cases n (%)	Controls n (%)
Ever Heard about FP?	Yes No	153 (99.4%) 1 (0.6%)	305 (99%) 3 (1%)
Source of Information (*n* = 458)	Health Workers Media Community Schools/Teachers Friends	135 (87.7%) 43 (27.9%) 54 (35%) 1 (0.6%) 0 (0%)	274 (89%) 125 (40.6%) 131 (42.5%) 17 (5.5%) 4 (1.3%)
Use of FP	Spacing Limiting Prevent Unwanted Pregnancy Prevent STI I have no idea	134 (87%) 36 (23.4%) 69 (44.8%) 12 (7.8%) 1 (0.6%)	271 (88%) 87 (28.2%) 200 (64.9%) 64 (20.8%) 3 (0.9%)
Which methods do you know? (*n* = 458)	Condoms Pills Injectables Implants IUD Natural Methods Permanent Methods	31 (20.3%) 116 (75.8%) 56 (36.6%) 123 (80.4%) 47 (30.7%) 18 (11.8%) 2 (1.3%)	139 (45.6%) 253 (83%) 138 (45.2%) 284 (93.1%) 153 (50.2%) 34 (11.1%) 2 (0.6%)
Where FP services found? (*n* = 458)	Health Center Health Post Hospital Private Clinics Pharmacies	117 (76.5%) 110 (71.9%) 38 (24.8%) 46 (30%) 4 (2.6%)	284 (93.1%) 218 (71.5%) 130 (42.6%) 162 (53.1%) 13 (4.3%)
Knowledge Status	Good Poor	45 (29.2%) 109 (70.8%)	169 (54.9%) 139 (45.1%)

### Discussion and decision related characteristics

More than half respondents in both cases and controls (61% and 59% respectively) discussed about family planning with their partner and above 80% respondents in both groups discussed about family planning with health professionals. Only 30 (19%) of cases and 103 (33%) of controls decided by themselves to use FP method, while the rest of the respondents were either depended on their husband’s decision or decided together with their husbands to use FP method. Less than half (42%) of respondents in the case group and inversely more than half (56%) of respondents in the control group were supported and approved by their spouses to use FP method (Table 4).

**Table 4:** Discussion and Decision related characteristics of reproductive age women in Dewa Chefa district, Ethiopia, March 29-April 25, 2021

Variables	Response Category	Cases n (%)	Controls n (%)
Discussion on FP with Spouse/Partner	Yes No	95 (61.7%) 59 (38.3%)	184 (59.7%) 124 (40.3%)
Frequency of Discussion (*n* = 279)	Once Twice Often	23 (24.2%) 19 (20%) 53 (55.8%)	51 (27.7%) 37 (20.1%) 96 (52.2%)
Decider of FP use	Self Husband Together	30 (19.5%) 75 (48.7%) 49 (31.8%)	103 (33.4%) 72 (23.4%) 133 (43.2%)
Support and Approval of Spouse to Use FP method	Yes No	66 (42.9%) 88 (57.1%)	175 (56.8%) 133 (43.2%)
Discussion on FP with Health Workers	Yes No	130 (84.4%) 24 (15.6%)	250 (81.2%) 58 (18.8%)
Visit of Health Facility in the last 6 months	Yes No	109 (70.8%) 45 (29.2%)	205 (66.6%) 103 (33.4%)

### Determinants of unmet need for family planning

After 21 categorical variables were entered to bivariable regression model and tested for association separately, 12 variables with p-value of less than 0.25 were screened and included in the final model. Then after, age of woman, having knowledge about FP, decider of FP use and support and approval of spouse for FP use maintained their association with unmet need for family planning.

Accordingly, respondents aged 35–49 years were found to be 6.6 times more likely to have unmet need for FP compared with women 15–19 years old [AOR = 6.6 (1.1–39)]. Respondents with poor knowledge on FP were found to be 1.9 times more likely to have unmet need for FP compared with women having good knowledge on FP [AOR = 1.9 (1.1–3.1)]. Respondents whose husbands decided their use of FP method were found to be 3.8 times more likely to have unmet need for FP compared with women decided by themselves [AOR = 3.8 (2.1–6.9)]. Respondents who decided together with their husbands on FP method use were found to be 2.3 times more likely to have unmet need for FP compared with women decided by themselves [AOR = 2.3 (1.07–5.1)]. Respondents whose husbands doesn’t supported and disapproved them to use FP were found to be 2.1 times more likely to have unmet need for FP compared with women whose husbands supported and approved them to use FP [AOR = 2.1 (1.08-4)] (Table 5).

**Table 5:** Determinants of unmet need for family planning (Bivariable and Multivariable logistics regression analysis result), Dewa Chefa district, Ethiopia, March 29-April 25, 2021

Variables		Response Category	Unmet Need for FP					COR (95% CI)	AOR (95% CI)		
			Yes		No						
Age		< 20 years 20–34 years 35–49 years	2 115 37		45 208 55			1 **12.4 (2.9–52.2)** ^***^ **15.1 (3.5–66.2)** ^***^	1 5.2 (0.9–28) **6.6 (1.1–39)** ^*^		
Residence		Semi-Urban Rural	22 132		65 243			1 1.6 (0.9–2.7)	1 0.8 (0.4–1.6)		
Respondents Educational Status		No formal education Read and write Primary Secondary College & Above	73 46 18 6 11		106 90 43 28 41			**2.6 (1.2–5.3)** ^**^ 1.9 (0.8-4) 1.56 (0.6–3.7) 0.79 (0.26–2.4) 1	0.5 (0.05–3.8) 0.5 (0.05–3.6) 0.6 (0.07–4.7) 0.5 (0.08–2.6) 1		
Partners Educational Status		No formal education Read and write Primary Secondary College & Above	67 59 12 3 13		104 112 24 17 51			**2.5 (1.2–4.9)** ^**^ **2 (1.04–4.1)** ^*^ 1.9 (0.78–4.9) 0.69 (0.17–2.7) 1	0.9 (0.1–6.6) 0.7 (0.1–5.2) 0.7 (0.1–5.7) 0.7 (0.08-6) 1		
Respondents Occupation		Housewife Farmer Merchant Student Daily Laborer Gov’t employee Private employee	95 21 22 2 3 10 1		152 37 33 47 4 29 6			1 0.9 (0.5–1.6) 1 (0.58–1.9) **0.06 (0.01–0.3)** ^***^ 1.2 (0.26–5.5) 0.55 (0.26–1.2) 0.27 (0.03–2.25)	1 0.9 (0.5–1.9) 1.2 (0.6–2.4) 0.2 (0.02–1.4) 0.8 (0.1-5) 0.5 (0.08–2.6) 0.4 (0.04–4.2)		
Family Size		< 5 5 and Above	58 96		146 162			**0.67 (0.45–0.9)** ^*^ 1	1.5 (0.5–3.7) 1		
Age at First Marriage		< 18 years 18 and above	74 80		107 201			**1.7 (1.1–2.6)** ^**^ 1	1.2 (0.7–1.9) 1		
Repetition of Marriage		Once Twice More than Twice	111 32 11		236 64 8			1 1 (0.6–1.7) **2.9 (1.1–7.5)** ^*^	1 0.8 (0.4–1.4) 2 (0.7-6)		
Number of Living Children		0 1–2 3–4 5 and above	5 56 51 42		56 112 70 70			**0.15 (0.05–0.4)** ^***^ 0.8 (0.5–1.4) 1.2 (0.7-2) 1	0.3 (0.06–1.4) 0.9 (0.3–2.6) 1.4 (0.7–2.6) 1		
Knowledge Status		Good Poor	45 109		169 139			1 **2.9 (1.9–4.5)** ^***^	1 **1.9 (1.1–3.1)** ^**^		
Decider of FP use		Self Husband Together	30 75 49		103 72 133			1 **3.6 (2.1-6)** ^***^ 1.3 (0.7–2.1)	1 **3.8 (2.1–6.9)** ^***^ **2.3 (1.07–5.1)** ^*^		
Support and Approval of Spouse to Use FP method		Yes No	66 88		175 133			1 **1.7 (1.1–2.6)** ^**^	1 **2.1 (1.08-4)** ^*^		

* significant at p 0.05 ** significant at p 0.01 *** significant at p 0.001

## Discussion

The main aim of this study was to assess for independent determinants/predictors of unmet need for FP at Dewa Chefa District. After assessing all variables by both bivariable and multivariable logistic regression models; age of woman, main decider of FP use, knowledge about FP and support and approval of spouse for FP use were found to have significant association with unmet need for FP.

In this study, age of the woman was found associated with unmet need for FP. Women with older ages (35–49) had higher unmet need compared to the younger ages [15–19]. This finding was consistent with the study conducted at Shire Enda-Sillasie, where unmet need level increased with age [20]. The national multi-level analysis also showed that women between 45 and 49 years were more than two times more likely to have unmet need than the younger one [15–19, 30]. Findings from EDHS 2019 also showed that utilization of FP methods declines as age increases [7]. This may be due to older women will have low perceived risk of pregnancy as they are near to menopause and infrequent sexual intercourse [29, 30] and comprehensive awareness and service packages focuses more on the younger ages [20].

Again, in line with this study, study conducted at Gonji Kolela confirmed association of age with unmet need. But the level of unmet need decreases as age increases [23]. The national study conducted on predictors of unmet need also dictated, as age increases by one year, the odds of having unmet need decreases by 20% [16]. This may be due to as age increases the maturity to decide on family size will increase [23] and younger ages may be hindered by stigma to use FP methods [29]. Most surveys also showed that unmet need for spacing decreases with age while unmet need for limiting increases, with slightly lower levels of unmet need among the oldest group of women [2]. Another studies conducted at North Gonja district of Ghana and Burundi were also in-line with the result [26, 29].

Having poor knowledge was another factor significantly associated with unmet need. Studies conducted at north Gonja district of Ghana and Burundi also supported the finding [26, 29]. This may be due to, as knowledge about FP increases unmet need level decreases as a result of increase in accessibility of FP information and services and as well as informed decision making will increase [29]. Increased awareness also leads to increased demand for fertility regulation, increase in favorable attitude towards FP and utilization too [19].

Have no support and disapproval of spouse for FP use was also independent predictor of unmet need for FP in this study. Moreover, woman whose husbands decided her use of FP and woman who decided together with her husband were both found to have unmet need than woman who decided by herself. In study conducted at Debre Birhan, support and approval of spouse for FP use were found 99.5% protective to unmet need [22]. Studies conducted in Dangila and Tiro Afeta district also showed consistent findings [18, 24]. This indicates that male involvement is a factor and influences women attitude and utilization of FP service and contraceptives [24]. Studies conducted in Africa countries (Burkina Faso and Cameroon) also supported the above finding [27, 32, 33]. A woman who have support and approval of husband for FP use were 48% protected against unmet need [27]. In most of developing African countries, like Ethiopia, even though a small proportion of women in a union adopt contraceptive practices without the concern of their husbands, the final decider and promoter of FP use is generally the man, where the liberty of the woman is low [27, 32].

### Limitation of the study

The current study is conducted on currently married reproductive age women of rural part, which cannot be generalized to other groups (like unmarried sexually active age group) and cannot compare the difference of study setting (i,e with urban setting). Additionally, interviewer-based bias may occur in some variables as data collectors were HEW’s and HW’s. Finally, as in most case control studies, there may be recall bias and causation cannot be established.

## Conclusion and recommendations

After controlling confounders in the final model; age of the woman, main decider of FP use, knowledge about FP, support and approval of spouse for FP use were found to have significant association with unmet need for FP. Thus, women empowerment and male involvement in the program, increase women knowledge and the freedom of informed choice on FP use, identify women with higher unmet need and address them with youth friendly service and other platforms and assure informed decision making of couples are recommended. Additionally, other researchers are recommended to conduct further researches on factors associated with unmet need for FP by including male respondents and test their attitude towards FP. Mixed study approaches with qualitative method (like FGD, KII, …), assessing for supply and delivery of FP methods in HF’s, assessing for competence of FP providers and quality of counseling are also encouraged.

## Data Availability

The datasets used and/or analyzed during the current study are available from the corresponding author on reasonable request.

## Abbreviations

AOR: Adjusted Odds Ratio
CI: Confidence Interval
COR: Crude Odds Ratio
EDHS: Ethiopian Demographic and Health Survey
FMoH: Federal Ministry of Health
FP: Family Planning
GTP II: Growth and Transformational Plan 2
HF: Health Facilities
HH: Household
IUD: Intra-Uterine Device
PHCU: Primary Health Care Unit
RAW: Reproductive Age Woman
SDGs: Sustainable Developmental Goals
SPSS: Statistical Packages for Social Sciences
STI: Sexually Transmitted Diseases
